# Phenotypic variation and genetic diversity assessment of proso millet (*Panicum miliaceum* L.) germplasm resources in northern Xinjiang

**DOI:** 10.3389/fpls.2026.1792533

**Published:** 2026-05-19

**Authors:** Zhao Yun, Liu Jun, Wang Hong‑Jin, Zhao Yang, Hu Xiangwei, Zaituniguli Kuerban, Wang Hui, Yang Baoyi, Feng Guojun, Nadeem Bhanbhro

**Affiliations:** 1 Crop Research Institute, Xinjiang Academy of Agricultural Sciences, Urumqi, Xinjiang, China; 2 Seed Industry Development Center of Xinjiang Uygur Autonomous Region, Urumqi, Xinjiang, China; 3 Sanya Institute, Hainan Academy of Agricultural Sciences, Sanya, China; 4 National Nanfan Research Institute, Chinese Academy of Agricultural Sciences, Sanya, China; 5 Biotechnology Research Institute, Chinese Academy of Agricultural Sciences, Beijing, China

**Keywords:** genetic diversity, genotype × environment interaction, germplasm evaluation, phenotypic variation, proso millet (*Panicum miliaceum* L.), trait-based breeding

## Abstract

Characterizing genetic diversity in proso millet (*Panicum miliaceum* L.) is essential for crop improvement and breeding programs. This study evaluated the genetic diversity, phenotypic variation, and population structure of 46 proso millet accessions using five qualitative and twelve quantitative traits across two growing seasons (2023-2024) in northern Xinjiang. Grain color showed the highest genetic diversity index (H′ = 2.064), while panicle type showed the lowest (H′ = 1.383). Green panicle color (84.78%), long panicle branch length (63.04%), lateral panicle type (71.74%), and yellow grain color (86.96%) were predominant. Grain yield (Y) and grain weight per panicle (GWP) exhibited the highest coefficients of variation (15.00% and 14.61%, respectively) and substantial genetic diversity indices (Y: H′ = 2.11; GWP: H′ = 2.12), indicating considerable selection potential. Grain yield was significantly positively correlated with grain weight per panicle (r = 0.901, *P < 0.01*) and main panicle length (r = 0.863, *P < 0.01*). Combined analysis of variance revealed highly significant genotypic effects for all agronomic traits, while genotype × environment interaction effects were non-significant for all traits, indicating stable genotype rankings across years. Broad-sense heritability estimates were very high for yield-related traits, with grain yield (H² = 0.998) and grain weight per panicle (H² = 0.996) demonstrating strong genetic control. Stepwise regression analysis identified grain weight per panicle and main panicle length as the primary yield predictors (R² = 0.91). Principal component analysis showed PC1 and PC2 explained 70.13% of total variation, representing vegetative vigor and yield capacity, respectively. Cluster analysis identified three groups with distinct trait profiles suitable for dual-purpose breeding, early-maturing cultivar development, and forage breeding, respectively. The evaluated germplasm possesses substantial genetic diversity, providing valuable resources for breeding parent selection in northern Xinjiang proso millet improvement programs.

## 1 Introduction

Proso millet (*Panicum miliaceum* L.), a member of the Poaceae family, has maintained its agricultural significance across diverse geographical regions as a climate-resilient cereal crop (Stevens et al., 2021; Dai et al., 2021). Archaeological evidence places its domestication approximately 10,000 years ago in northern China (He et al., 2021), and traditional cultivars such as ‘Taromi’ remain integral to local farming systems in Xinjiang, where the crop constitutes a staple among the Kazakh population (Zhang et al., 2021). The crop’s exceptional drought tolerance, short growing season (60–90 days), and minimal water requirements (300–400 mm annually) make it strategically important for sustainable agriculture in semi-arid and arid environments (James et al., 2025; Zhao et al., 2025; Loni et al., 2023), which provides comparative context from foxtail millet, a closely related Setaria italica species sharing similar arid-zone adaptations). These attributes are particularly relevant in the context of global climate change and increasing food security concerns in marginal lands where conventional cereals struggle to thrive (Bhanbhro et al., 2024; 2025).

The adaptability of proso millet is evidenced by its recent expansion into marginal environments such as northern Xinjiang (Pavithra and Rawat, 2024; Luo et al., 2022), where the planting area reached approximately 13,333 ha, with average yields of 3.75 t·ha^-^¹. The successful establishment of a complete local value chain, encompassing cultivation, processing, and marketing, underscores the crop potential to contribute to food security and climate-resilient agriculture, as discussed in this chapter (Pavithra and Rawat, 2024; Nandini et al., 2026). This growth occurs within a challenging agricultural environment characterized by extreme continental climate conditions, including significant temperature fluctuations, limited precipitation, soil salinity, and relatively short frost-free periods (Han et al., 2020; Bhanbhro et al., 2020; Wimalasiri et al., 2023). Despite the crop’s inherent adaptability, the industry advancement is constrained by critical deficiencies in its genetic foundation: inadequate germplasm characterization, incomplete collection efforts, and insufficient evaluation protocols (AbdElgawad et al., 2020; Aibai et al., 2024). These limitations hinder targeted varietal improvement and sustainable development of the regional proso millet sector.

Germplasm resources encompassing landraces, improved varieties, wild relatives and genetic stocks constitute the fundamental basis for crop improvement and genetic research (Zhang et al., 2021). They serve as essential repositories of genetic diversity, harboring valuable alleles for key traits such as drought tolerance, disease resistance, early maturity and enhanced grain quality (Zhao et al., 2025). The systematic evaluation and characterization of these collections are critical prerequisites for understanding genetic potential, identifying superior genotypes, and developing climate-resilient cultivars (Mukami et al., 2019; Rivero et al., 2022). In northern Xinjiang, such assessment is particularly important due to the region’s distinctive agro-climatic pressures (Ma et al., 2025a; 2025b) and the urgent need to identify genotypes optimized for local agricultural systems. Furthermore, genotype × environment (G×E) interactions play a critical role in determining crop performance, making location-specific evaluation indispensable for maximizing agricultural productivity (Pei et al., 2023; Rizwan et al., 2025).

Recently studies on proso millet germplasm from Xinjiang and neighboring regions have reported substantial phenotypic variation, with coefficients of variation ranging from 12.37% to 91.40% and genetic diversity indices of 0.410 to 0.809 (Lv et al., 2025). Principal component analysis has revealed that the first four components account for 76.64% of total phenotypic variation, with yield per plant, stem diameter, effective panicle number, and leaf width identified as primary sources of variability. Cluster analysis based on morphological data has revealed distinct genetic differentiation patterns correlating with geographical origin in related millet species (Darkwa et al., 2020). However, critical knowledge gaps remain regarding the adaptive mechanisms, phenotypic stability, and precise yield-formation relationships of proso millet genotypes under northern Xinjiang conditions.

This study addresses these gaps through integrated evaluation of 46 cultivated and introduced proso millet accessions adapted to northern Xinjiang conditions using a multidisciplinary approach combining field-based agronomic characterization with multivariate statistical analysis. The specific objectives were to: (1) evaluate genetic diversity and phenotypic variation across qualitative and quantitative traits; (2) analyze genotype × environment (G×E) interactions and estimate broad-sense heritability for key agronomic traits; (3) develop optimized regression models linking grain yield with contributing traits; and (4) identify superior germplasm with desirable trait combinations for targeted breeding applications. The outcomes will provide empirical foundations for conservation strategies, germplasm selection, parental line identification, and breeding program design in support of the regional proso millet industry (Allier et al., 2020).

## 2 Materials and methods

### 2.1 Experimental materials

**Figure 1:**
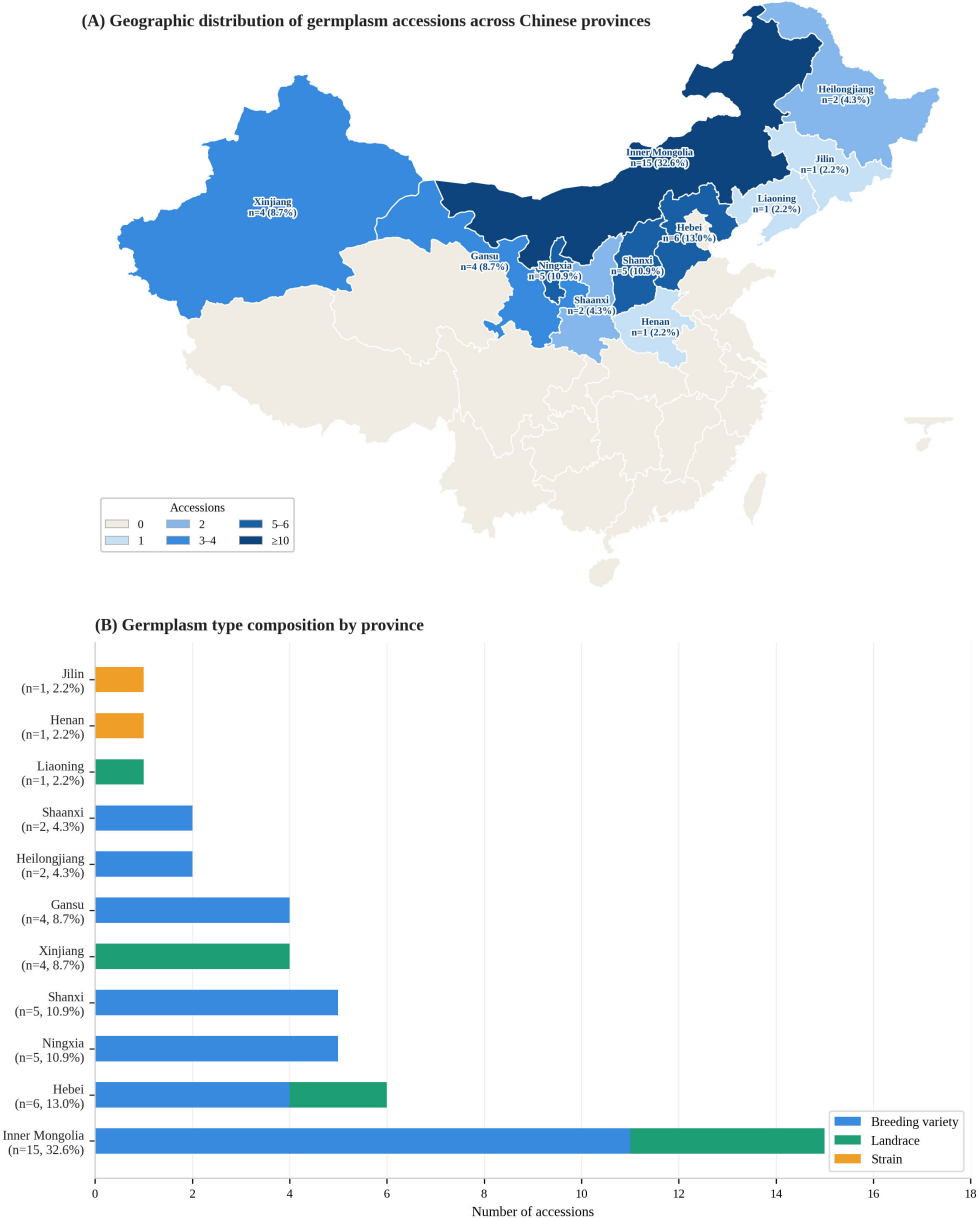
Geographic distribution of 46 proso millet (*Panicum miliaceum* L.) germplasm accessions across 11 Chinese provinces. **(A)** Map of China showing provinces of origin. Shading intensity reflects the number of accessions per province; provinces with collections are labeled with name, accession count (n), and percentage of the total collection. **(B)** Stacked bar chart showing the composition of germplasm types (breeding variety, landrace, strain) per province.

This study evaluated 46 proso millet accessions collected from 11 provinces and autonomous regions of China (Inner Mongolia, Gansu, Shanxi, Ningxia, Hebei, Xinjiang, Heilongjiang, and Shaanxi, Liaoning, Henan, Jilin; **Figure 1**). Accessions were selected to represent diverse ecological zones across major proso millet-producing regions of China (**Supplementary Table 1**). A total of 17 phenotypic traits were evaluated: five qualitative traits (inflorescence color, panicle branch length, inflorescence density, panicle type, and grain color) and 12 quantitative traits (growth period, number of productive tillers, main stem height, main panicle length, number of main stem nodes, main stem diameter, flag leaf length, flag leaf width, number of leaves on the main stem, grain weight per panicle, thousand-grain weight, and grain yield).

### 2.2 Experimental design

The field trial was conducted over two consecutive growing seasons (2023 and 2024) in Qitai County, Xinjiang, China (44.02°N, 89.57°E). The experiment employed a randomized complete block design (RCBD) with three biological replicates. Each experimental plot measured 8 m² (5 m × 1.6 m) and was planted using a locally adapted plastic film mulching system with hole-seeding at 40 cm row spacing, 10 cm hill spacing and a seeding rate of approximately 6.7 g per plot (equivalent to 8.4 kg·ha^-^¹), calculated based on a target plant density of 375,000 hills ha^-^¹ (40 cm × 10 cm spacing) with 4 seeds per hill and the mean 1000-grain weight of 8.41 g of the accessions evaluated. This seed rate, although lower than conventional broadcast seeding rates, is appropriate for the hole-seeding plastic film mulching system used in this study, which achieves higher germination efficiency per hill than broadcast methods (Palchetti et al., 2023). Diammonium phosphate (150 kg ha^-^¹) was applied as basal fertilizer. All plots received uniform irrigation four times during the growth cycle, and standardized field management practices were maintained throughout. Border rows surrounded the experimental area to minimize edge effects.

### 2.3 Trait evaluation methods

#### 2.3.1 Qualitative trait assessment

Five qualitative traits were visually evaluated following the descriptors and data standards for common millet germplasm (Ren et al., 2024): inflorescence color (green/purple), panicle branch length (referring to the length of primary branches arising from the main panicle axis, classified as short: <5 cm, medium: 5–10 cm, and long: >10 cm), inflorescence density (sparse/medium/moderately dense/dense), panicle type (lateral/lax/compact), and grain color (yellow/red/brown/white/gray/variegated). Ten representative plants per plot were assessed for each trait.

#### 2.3.2 Quantitative trait measurement

Twelve quantitative traits were measured following standard descriptors (Anyaoha et al., 2023; Zhao et al., 2025), with ten representative plants per plot evaluated for each trait. Growth period (GP, days) was recorded as the number of days from seedling emergence to physiological maturity. Number of productive tillers (NPT, tillers plant^-^¹) was counted at maturity, defined as all tillers bearing a panicle. Main stem height (MSH, cm) was measured from the soil surface to the base of the main panicle at maturity. Main panicle length (MPL, cm) was measured from the basal node of the main panicle to its tip. Number of main stem nodes (NMSN) was counted on the main culm at maturity. Main stem diameter (MSD, mm) was measured at the first internode above the ground using digital calipers. At the heading stage, flag leaf length (LL, cm) was measured from the ligule to the leaf tip, and flag leaf width (LW, mm) was measured at the widest point of the flag leaf blade using digital calipers. Number of leaves on the main stem (NL) was counted at maturity. Grain weight per panicle (GWP, g) was determined by threshing and weighing grains from the main panicle using a precision balance. Thousand-grain weight (GW, g) was determined by counting 1,000 clean, air-dried grains and recording their weight using a precision balance. Grain yield (Y, kg·ha^-^¹) was calculated from plot harvest weight adjusted to 13% moisture content, then converted to a per-hectare basis. All measurements were performed by the same trained personnel using calibrated instruments to minimize inter-observer variation.

### 2.4 Data analysis

#### 2.4.1 Genetic diversity analysis

Genetic diversity of qualitative traits was assessed using the Shannon-Weaver diversity index: H′ = –Σ(pi × ln pi), where pi represents the phenotypic frequency of category i (Gebre et al., 2025). Quantitative trait variation was evaluated using the coefficient of variation: CV = (standard deviation/mean) × 100% (Pélabon et al., 2020).

#### 2.4.2 Multivariate statistical analysis

Principal component analysis (PCA) was performed using SPSS software (Version 26.0, IBM Corp.) (Elhaik, 2022; Greenacre et al., 2022; Varraso et al., 2012). Comprehensive evaluation scores (F-values) for each accession were calculated based on PCA results using weighted principal component contributions. Pearson correlation analysis and hierarchical cluster analysis (Ward’s method with squared Euclidean distance) were conducted using Origin 2024 software.

#### 2.4.3 Combined analysis of variance and heritability

A mixed-effects model was used to analyze the two-year data, with year (environment) treated as a random effect and genotype as a fixed effect. Combined analysis of variance (ANOVA) was performed to partition phenotypic variance into genotypic (σ²g), genotype × environment interaction (σ²ge), and residual error (σ²e) components. Broad-sense heritability was estimated as: H² = σ²g/(σ²g + σ²ge/e + σ²e/er), where e is the number of environments and r is the number of replicates (Yu et al., 2022). Multiple comparisons were performed using the LSD method (*p < 0.05*).

#### 2.4.4 Regression analysis

Stepwise multiple regression analysis was performed using SPSS 26.0 to identify the most significant predictors of grain yield. Grain yield was defined as the dependent variable and the twelve quantitative traits were defined as independent variables. Variables were entered and removed at significance thresholds of *p ≤ 0.05* and *p ≥ 0.10*, respectively. Model fit was assessed using the coefficient of determination (R²), adjusted R², and the standard error of the estimate.

## 3 Results

### 3.1 Genetic diversity of qualitative traits

**Figure 2:**
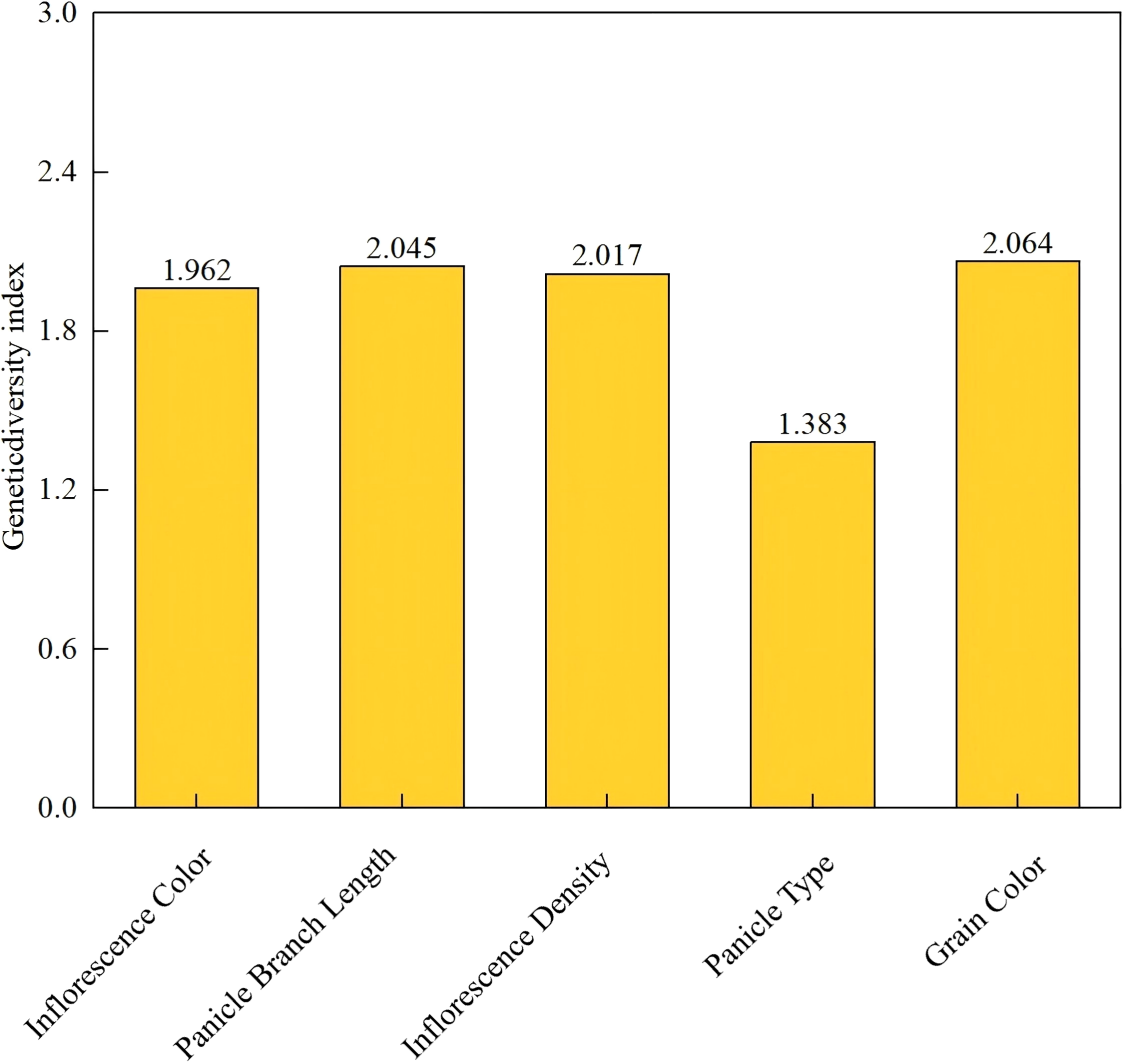
Shannon-Weaver genetic diversity index (H′) for five qualitative traits in proso millet germplasm. Grain color exhibited the highest diversity (H′ = 2.064), while panicle type showed the lowest (H′ = 1.383).

The genetic diversity of five qualitative traits across 46 proso millet accessions is presented in **Figure 2**. Grain color exhibited the highest genetic diversity index (H′ = 2.064), indicating broad variation and stable genetic expression for this trait, followed by panicle branch length (H′ = 2.045). Panicle type displayed the lowest diversity index (H′ = 1.383). The genetic diversity indices for the five qualitative traits ranked as follows: grain color > panicle branch length > inflorescence density > inflorescence color > panicle type.

### 3.2 Distribution patterns of qualitative traits

**Figure 3:**
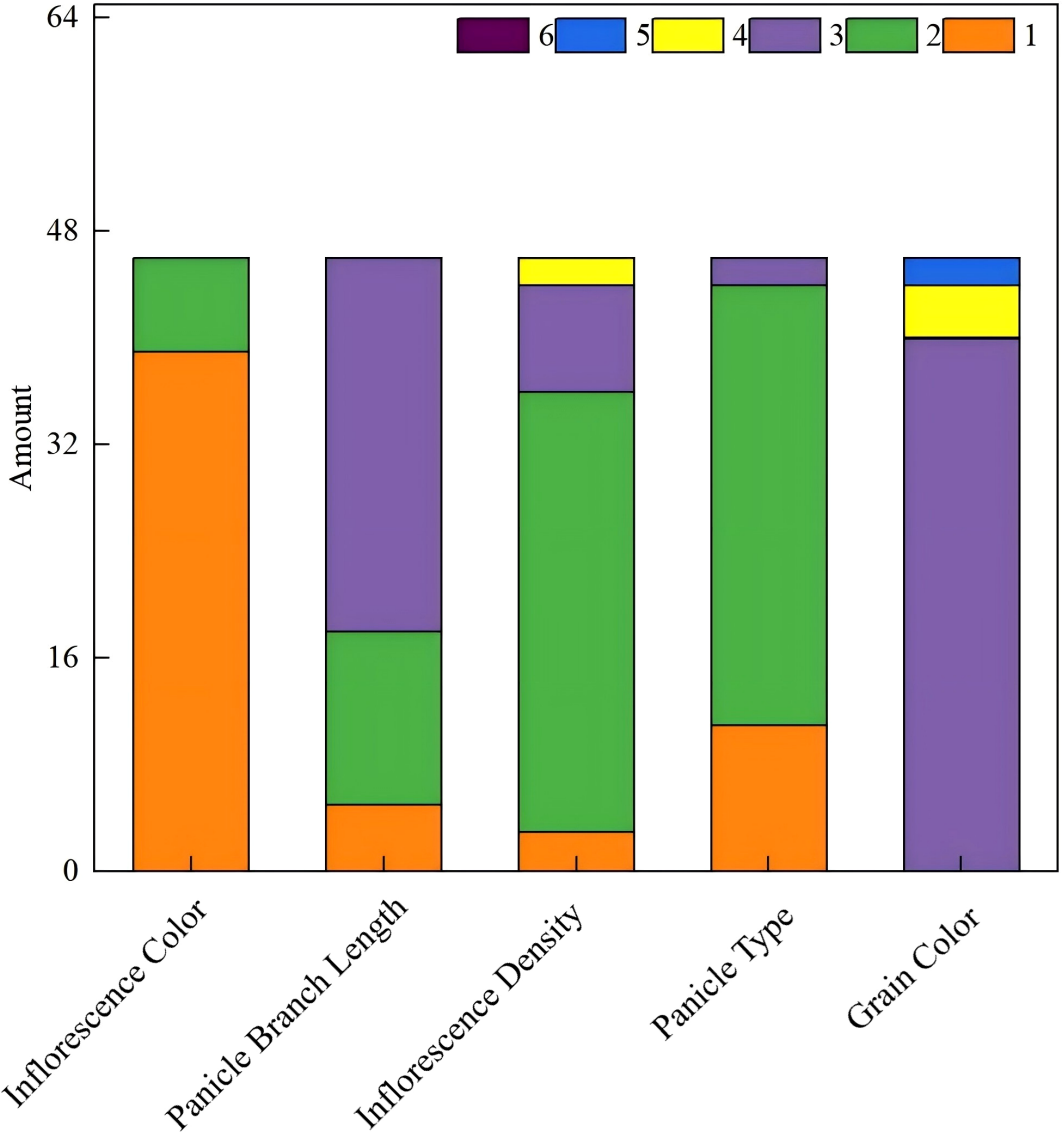
Frequency distribution of five qualitative traits in proso millet germplasm. Inflorescence Color: 1. Green, 2. Purple; Panicle Branch Length: 1. Short, 2. Medium, 3. Long; Inflorescence Density: 1. Sparse, 2. Medium, 3. Moderately dense, 4. Dense; Panicle Type: 1. Lax panicle, 2. Lateral panicle, 3. Compact panicle; Grain Color: 1. White, 2. Gray, 3. Yellow, 4. Red, 5. Brown, 6. Variegated.

The distribution patterns and frequency analysis of the five qualitative traits revealed distinct variation within the germplasm collection (**Figure 3**). Inflorescence color exhibited a clear predominance of green types (84.78%), with purple types representing a minority variant (15.22%). Purple inflorescence pigmentation was observed in the same accessions across both growing seasons (2023 and 2024), confirming that this trait is under stable genetic control in this germplasm panel. Although anthocyanin expression can be influenced by environmental factors such as day length and nitrogen availability (Zhang et al., 2026), no switching between pigmentation classes was observed across years in this study. Panicle branch length showed a skewed distribution toward longer types (63.04%), followed by medium (28.26%) and short (8.70%) types. Inflorescence density was predominantly medium (71.74%), with slightly dense types (15.22%), sparse types (8.70%), and dense types being uncommon (4.35%). Panicle type was dominated by lateral panicles (71.74%), with lax panicles as a secondary type (23.91%) and compact panicles being relatively uncommon (4.35%) (**Table 1**). Grain color was strongly dominated by yellow types (86.96%), with red and brown grain types representing 8.70% and 4.34%, respectively; white, gray, and variegated grain types were absent from this collection. These distribution patterns provide valuable baseline information for germplasm management and targeted breeding strategies.

**Table 1:** Classification and coding system for five qualitative traits in proso millet germplasm.

Trait	Descriptive criteria
Inflorescence Color	Green = 1, Purple = 2
Panicle Branch Length	Short = 1, Middle = 2, Long = 3
Inflorescence Density	Sparse = 1, Middle = 2, Slightly dense = 3, Dense = 4
Panicle Type	Scattered = 1, Side = 2, Dense = 3
Grain Color	White = 1, Grey = 2, Yellow = 3, Red = 4, Brown= 5, Broken = 6

### 3.3 Variation and diversity of quantitative traits

Analysis of 12 quantitative traits revealed considerable variation in both coefficient of variation (CV) and Shannon–Weaver diversity index (H′) across the 46 accessions (**Table 2**). Leaf width (LW) exhibited the highest CV (24.14%), indicating substantial variation in this trait among accessions. Grain yield (Y) showed the second highest CV (15.00%), followed by number of main stem nodes (NMSN, CV = 14.85%), main stem diameter (MSD, CV = 14.81%), number of leaves (NL, CV = 14.67%), and grain weight per panicle (GWP, CV = 14.61%), with GWP ranging from 18.96 to 36.88 g. Main stem height (MSH) showed a CV of 13.88%, and main panicle length (MPL) a CV of 13.83%. Moderate variation was observed for leaf length (LL, CV = 10.04%), thousand-grain weight (GW, CV = 10.05%), and number of productive tillers (NPT, CV = 8.92%), while growth period (GP) showed the lowest CV (8.23%), reflecting relatively stable phenological development across accessions.

**Table 2:** Mean values, ranges, amplitude, coefficients of variation (CV), and Shannon–Weaver diversity indices (H′) for 12 quantitative traits in 46 proso millet germplasm accessions (combined 2023–2024).

Trait	Mean ± SD	Range	Amplitude	CV (%)	H′
Growth Period (GP/d)	98.61 ± 8.11	76.67–116.00	39.33	8.23	1.956
No. of Productive Tillers (NPT)	3.36 ± 0.30	2.80–4.20	1.4	8.92	1.815
Main Stem Height (MSH/cm)	188.33 ± 26.14	114.35–218.71	104.36	13.88	1.788
Main Panicle Length (MPL/cm)	37.72 ± 5.22	25.26–46.19	20.93	13.83	2.023
No. of Main Stem Nodes (NMSN)	7.61 ± 1.13	4.12–9.46	5.34	14.85	1.865
Main Stem Diameter (MSD/mm)	7.10 ± 1.05	4.03–8.60	4.58	14.81	1.984
Leaf Length (LL/cm)	36.33 ± 3.65	28.00–41.37	13.38	10.04	2.155
Leaf Width (LW/cm)	1.75 ± 0.42	0.80–2.72	1.92	24.14	2.11
No. of Leaves (NL)	8.16 ± 1.20	4.45–9.93	5.48	14.67	1.849
Grain Weight per Panicle (GWP/g)	28.11 ± 4.11	18.96–36.88	17.92	14.61	2.122
1000-Grain Weight (GW/g)	8.19 ± 0.82	6.18–9.70	3.52	10.05	2.126
Yield (Y/kg·hm^-^²)	4143.67 ± 621.75	2840.24–5513.26	2673.02	15	2.11

Shannon–Weaver diversity indices ranged from 1.788 (MSH) to 2.155 (LL). Leaf length demonstrated the highest H′ value (2.155), followed by thousand-grain weight (H′ = 2.126), grain weight per panicle (H′ = 2.122), leaf width and grain yield (both H′ = 2.11). Main panicle length showed intermediate diversity (H′ = 2.023), while main stem diameter (H′ = 1.984), growth period (H′ = 1.956), number of main stem nodes (H′ = 1.865), number of leaves (H′ = 1.849), number of productive tillers (H′ = 1.815), and main stem height (H′ = 1.788) showed comparatively lower diversity levels. It should be noted that the Shannon–Weaver diversity index (H′) is non-linear and sensitive to the number of phenotypic classes; therefore, comparisons of H′ values across traits with differing numbers of categories should be interpreted with appropriate caution. Furthermore, since approximately 59% of the 46 accessions originate from only three provinces (Inner Mongolia, Gansu, and Xinjiang), the observed diversity indices reflect the genetic composition of this specific collection rather than the full breadth of Chinese proso millet diversity. Accordingly, conclusions regarding diversity levels should be considered within the context of this geographically structured sample.

### 3.4 Phenotypic distribution of quantitative traits

**Figure 4:**
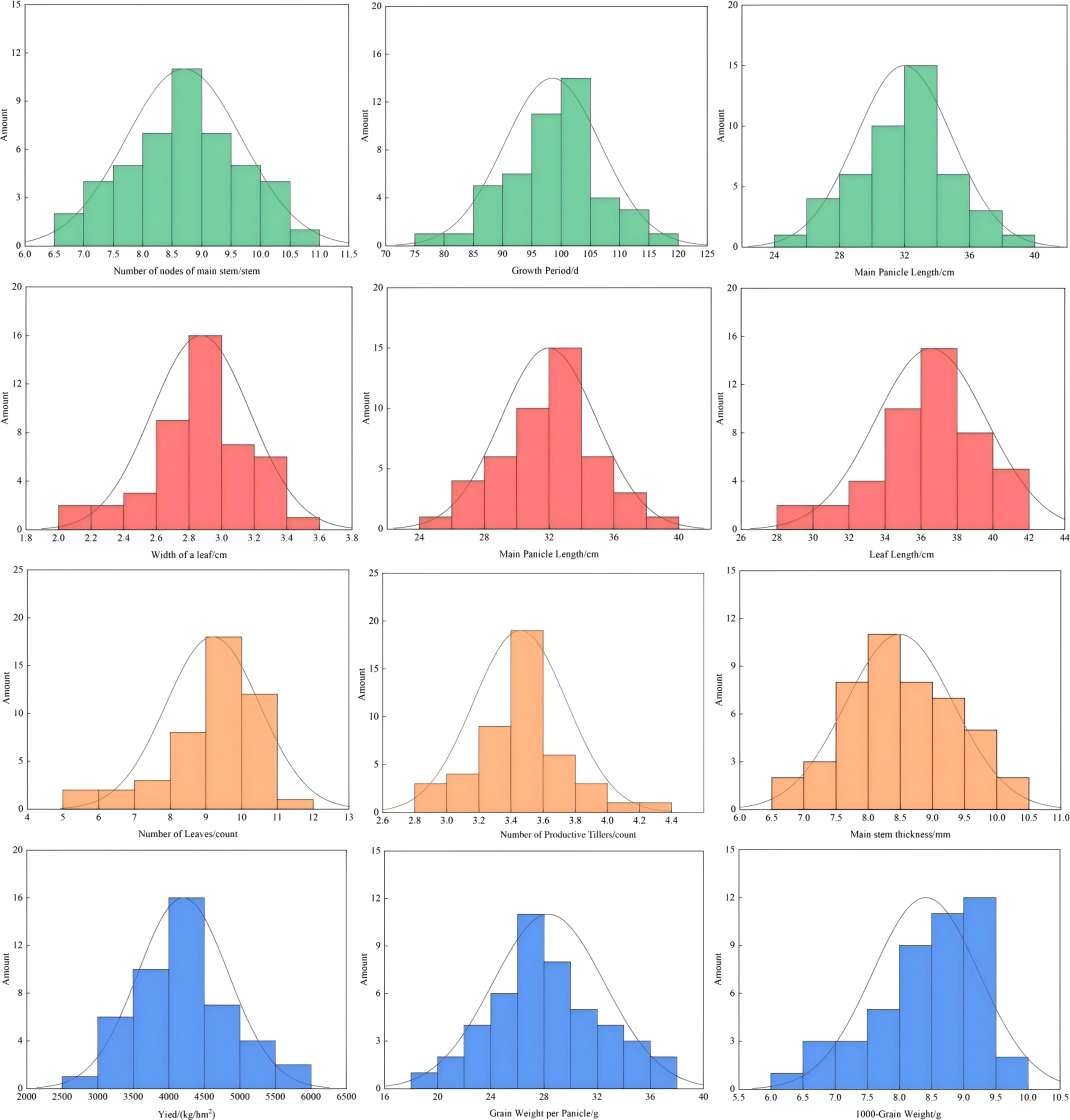
Frequency distribution and phenotypic variation of 12 quantitative traits in proso millet germplasm. Histograms show the distribution patterns with normal distribution curves overlaid. Traits displayed include: growth period (GP, days), main stem height (MSH, cm), main panicle length (MPL, cm), number of main stem nodes (NMSN), main stem diameter (MSD, mm), leaf length (LL, cm), leaf width (LW, mm), number of productive tillers (NPT), number of leaves (NL), grain weight per panicle (GWP, g), thousand-grain weight (GW, g), and grain yield (Y, kg·ha^-^¹).

Phenotypic variation analysis conducted on 12 agronomic traits across the 46 accessions indicated continuous variation for all traits with wide ranges and substantial phenotypic diversity (**Figure 4**). Growth period ranged from 75 to 110 days, with 78.26% of accessions concentrated between 85 and 105 days; a few late-maturing genotypes extended up to 110 days, indicating the presence of photoperiod-sensitive materials that may be valuable for specific agro-ecological zones. Main stem height showed a positively skewed distribution, with the majority of accessions classified as tall-stature types, reflecting the predominantly tall-growing ecotype characteristic of northern Chinese proso millet germplasm. Main panicle length ranged from 24 to 36 cm, with 82.61% of accessions distributed between 22 and 36 cm and 17.39% classified as long-panicle types (≥36 cm); given the strong positive correlation of main panicle length with grain yield, these long-panicle accessions represent priority targets for yield-oriented breeding. Main stem diameter ranged from 4.5 to 9.0 mm, with thick-stem types (≥9.0 mm) accounting for 4.35% of the collection, representing valuable resources for lodging-resistant cultivar development. The number of productive tillers was dominated by the 3-tiller type (73.91%), a structure favoring assimilate concentration in the main panicle. High-yielding accessions (≥4,500 kg·ha^-^¹) accounted for 13.04% of the collection, indicating significant potential for further yield enhancement through targeted crossing and genotype aggregation strategies.

### 3.5 Correlation analysis of quantitative traits

**Figure 5:**
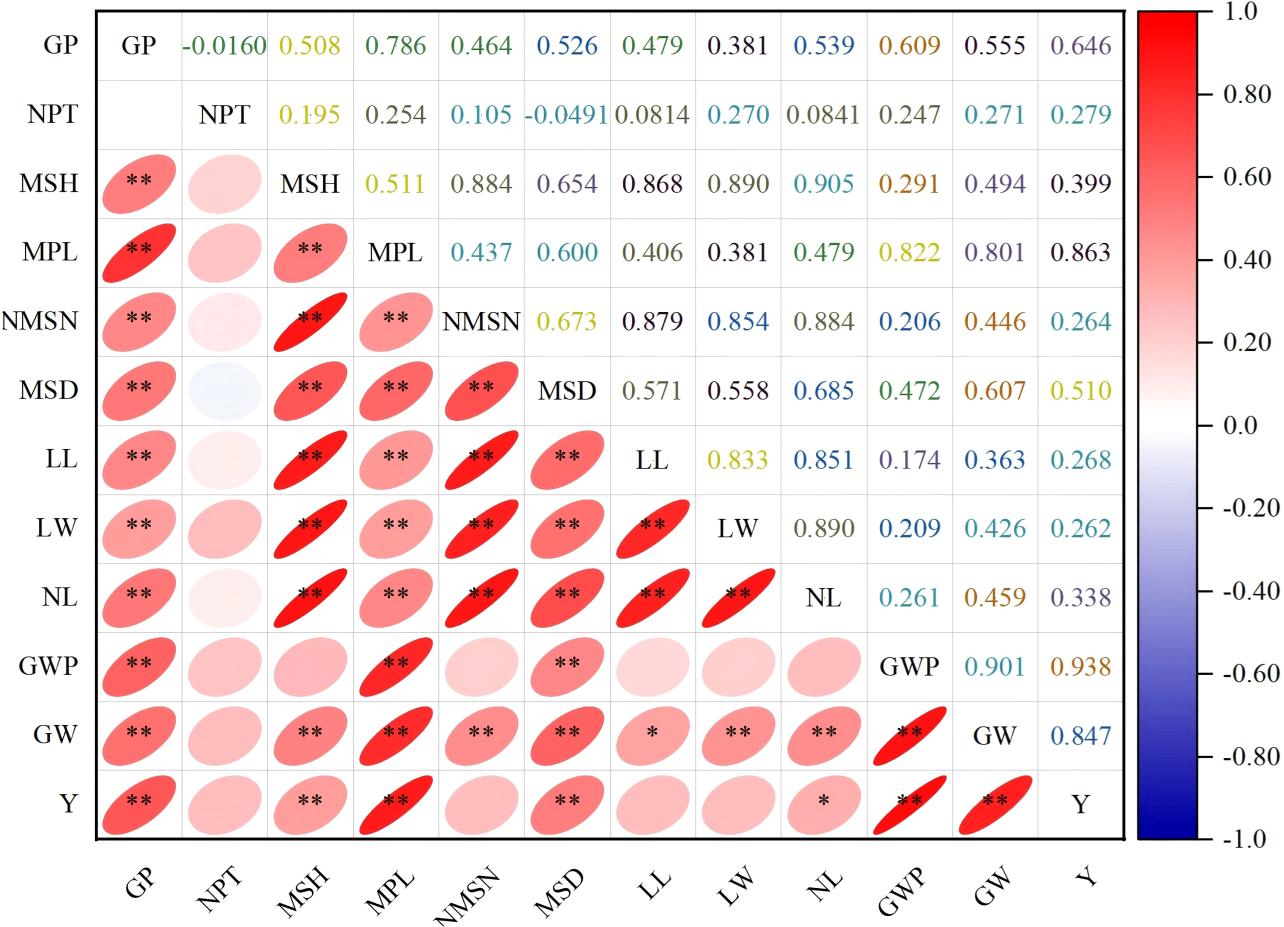
Pearson correlation coefficients among quantitative traits in proso millet germplasm. The lower triangle shows correlation ellipses with significance levels (*P < 0.05*; **P < 0.01), and the upper triangle displays correlation coefficient values. Color intensity indicates correlation strength (red: positive; blue: negative). GP, growth period; NPT, number of productive tillers; MSH, main stem height; MPL, main panicle length; NMSN, number of main stem nodes; MSD, main stem diameter; LL, leaf length; LW, leaf width; NL, leaf number; GWP, grain weight per panicle; GW, thousand-grain weight; Y, yield.

Correlation analysis of key agronomic traits revealed that grain yield (Y) was highly significantly positively correlated with grain weight per panicle (GWP) and main panicle length (MPL) (r =0.938 and 0.863, respectively; *P < 0.01*), confirming that large-panicle characteristics constitute the primary yield-formation pathway in this germplasm under the prevailing field conditions of this study (**Figure 5**). Grain weight per panicle also showed a strong positive correlation with thousand-grain weight (GW) (r = 0.847, *P < 0.01*), suggesting that sink capacity expansion in this germplasm operates through concurrent increases in both spikelet number per panicle and individual grain mass, two complementary components that breeders can exploit simultaneously. Importantly, thousand-grain weight was also significantly positively correlated with grain yield (r = 0.847, *P < 0.01*), reinforcing its value as a secondary selection criterion for yield improvement. Main stem height (MSH) exhibited strong positive correlations with main stem node number (NMSN) and main stem diameter (MSD) (r ≥ 0.884, *P < 0.01*) but showed a weak positive correlation with number of productive tillers (NPT) (r = 0.195, not significant), reflecting a plant architecture that prioritizes main stem growth over tillering-an adaptive strategy for concentrating photoassimilates in the main panicle under semi-arid conditions. Leaf length (LL) was highly significantly positively correlated with leaf width (LW) (r = 0.833, *P < 0.01*) and showed moderate positive correlations with main panicle length and grain weight per panicle (r ≈ 0.36–0.41, *P < 0.05*), indicating a modest contribution of flag leaf area to grain filling capacity. Growth period (GP) exhibited weak correlations with most yield traits (r < 0.20), suggesting that early-maturing genotypes can be selected without substantial yield penalty, a particularly valuable finding for breeding short-season adapted cultivars.

### 3.6 Principal component analysis

Principal component analysis (PCA) was applied to the 12 quantitative traits to identify the major axes of phenotypic variation among the 46 accessions (**Table 3**). The first four principal components collectively explained 86.69% of total phenotypic variation. PC1 accounted for the largest proportion of variance (45.97%), with high positive loadings for main stem height (0.400), number of main stem nodes (0.403), main stem diameter (0.388), and number of leaves (0.395), representing a “Vegetative Vigor” factor that captures variation in overall plant architecture and biomass accumulation. PC2 contributed 24.16% of variance and was dominated by grain weight per panicle (0.570), thousand-grain weight (0.550), and grain yield (0.560), clearly representing a “Yield Capacity and Grain Size” axis that distinguishes high-yielding accessions based on sink strength and grain filling efficiency. PC3 accounted for 9.12% of variance, with high positive loadings for leaf length (0.578) and main panicle length (0.503), representing a “Panicle and Leaf Architecture” factor that reflects morphological adaptation related to light interception and photosynthetic capacity. PC4 contributed 7.44% of variance and was dominated by growth period (0.837), representing a “Phenological Maturity” axis that separates early- from late-maturing genotypes. Together, PC1 and PC2 explained 70.13% of total variation, confirming that vegetative vigor and yield components are the two dominant dimensions of diversity in this collection.

**Table 3:** Principal component analysis (PCA) of 12 quantitative traits in 46 proso millet germplasm accessions: component loadings, eigenvalues, and variance contribution rates (combined 2023–2024 data).

Trait	PV1	PV2	PV3	PV4
Growth Period	0.131	0.188	0.271	0.837
No. of Productive Tillers	-0.322	-0.019	0.403	-0.064
Main Stem Height	0.400	0.016	0.120	-0.173
Main Panicle Length	0.314	-0.105	0.503	0.051
No. of Main Stem Nodes	0.403	-0.001	-0.194	-0.116
Main Stem Diameter	0.388	0.039	-0.199	0.056
Leaf Length	0.290	-0.055	0.578	-0.268
Leaf Width	0.259	-0.081	-0.218	0.345
Number of Leaves	0.395	0.008	-0.171	-0.155
Grain Weight per Panicle	0.016	0.570	-0.023	-0.044
1000-Grain Weight	0.009	0.550	0.091	-0.164
Yield	0.000	0.560	-0.012	-0.042
Eigenvalue	5.639	2.964	1.119	0.912
Variance contribution (%)	45.969	24.160	9.123	7.435
Cumulative contribution (%)	45.969	70.129	79.252	86.687

Comprehensive evaluation scores (F-values) based on weighted PC contributions were calculated to rank the 46 accessions by overall agronomic performance (**Table 4**). The top-ranked accession was Yishu 13 (F = 1.785), followed by Chishu 5 (F = 1.404), Yanshu 11 (F = 1.304), Gumi 23 (F = 1.288), and Yimi 21 (F = 1.267). Among the top 10 ranked accessions, the majority originated from Inner Mongolia and Xinjiang, with several accessions from Gansu and Ningxia also represented. These top-performing genotypes combine superior yield potential with favorable vegetative architecture, making them priority candidates for direct utilization in breeding programs. Conversely, the lowest-ranked accessions included Habahe 2 (F = –3.799), Jinshu 10 (F = –2.871), and Tuoli 1 (F = –1.407), which were characterized by reduced yield components and shorter growth periods, reflecting alternative adaptive strategies suited to specific agro-ecological niches.

**Table 4:** Comprehensive evaluation scores (F-values) and ranking of all 46 proso millet germplasm accessions based on weighted principal component analysis of 12 quantitative traits (combined 2023–2024 data).

Rank	No.	Variety	F-value	Rank	No.	Variety	F-value
1	21	Yishu 13	1.785	24	6	Ningmi 10	0.176
2	28	Chishu 5	1.404	25	15	Yimi 11	0.127
3	29	Yanshu 11	1.304	26	13	Shanmi 2	0.121
4	4	Gumi 23	1.288	27	37	Longmi 19	0.109
5	18	Yimi 21	1.267	28	41	N1255-2-3	0.033
6	24	Neimi 5	1.259	29	31	Jinshu 8	0.032
7	35	Longmi 15	1.258	30	46	Jishu 8	-0.001
8	19	Yishu 10	1.094	31	44	Jishu 4	-0.245
9	17	Yimi 16	1.091	32	39	N1524-3	-0.249
10	22	Yishu 15	1.056	33	7	Ningmi 17	-0.252
11	20	Yishu 11	1.035	34	42	Jishu 9	-0.273
12	23	Yixuanhuangmi	0.923	35	25	Chimi 3	-0.282
13	16	Yimi 12	0.922	36	34	Longmi 14	-0.344
14	12	Shanmi 1	0.896	37	26	Chishu 9	-0.429
15	5	Gumi 24	0.805	38	3	Gumi 22	-0.464
16	30	Yanshu 13	0.745	39	1	Qishu 1	-1.038
17	14	Yimi 5	0.656	40	38	N1278-1-1	-1.131
18	45	Jishu 7	0.544	41	2	Qishu 3	-1.187
19	43	Jishu 3	0.536	42	10	Tuoli 1	-1.407
20	40	N1191-2-1-3	0.350	43	33	Jinshu 10	-2.871
21	36	Longmi 17	0.341	44	8	Habahe 2	-3.799
22	11	Yilizao 1	0.318	45	32	Jinshu 9	-3.824
23	27	Chishu 3	0.271	46	9	Habahe 1	-3.951

### 3.7 Cluster analysis

**Figure 6:**
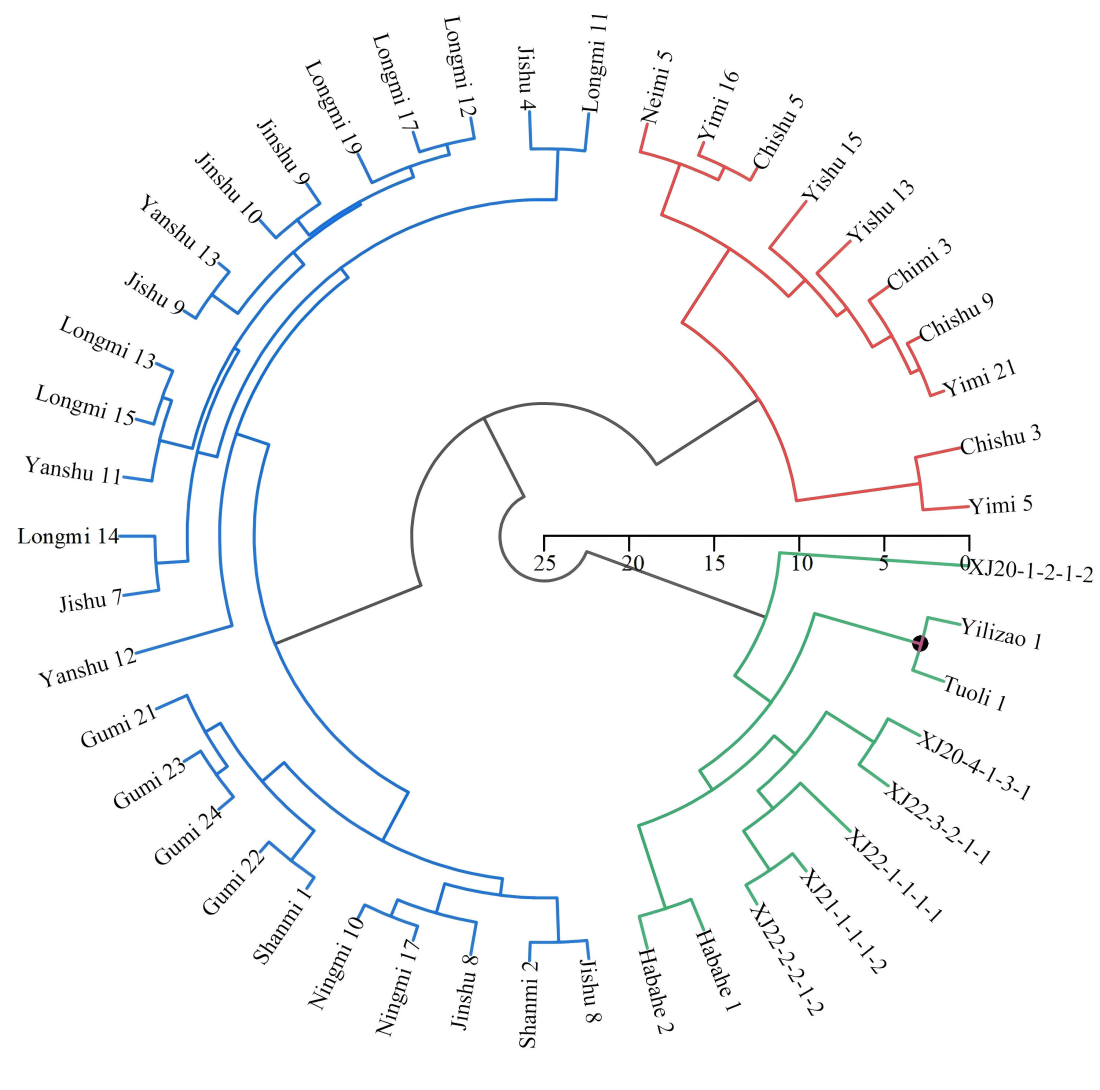
Hierarchical cluster analysis of proso millet germplasm based on quantitative trait variation. UPGMA clustering with euclidean distance grouped 46 accessions into three clusters: Group I (21.74%, red branches) with superior yield traits; Group II (21.74%, blue branches) with early maturity and lodging resistance; and Group III (56.52%, green branches) with potential for forage breeding.

Hierarchical cluster analysis (UPGMA method, squared Euclidean distance) based on the 12 quantitative traits classified the 46 accessions into three phenotypically and functionally distinct groups at a Euclidean distance threshold of 10 (**Figure 6**).

Group I contained 10 accessions (21.74%) representing the elite high-yield cluster, with mean grain weight per panicle (34.26 g), thousand-grain weight (9.23 g), and grain yield (5,112.90 kg·ha^-^¹) all significantly exceeding the overall population averages of 28.09 g, 8.18 g, and 4,143.94 kg·ha^-^¹, respectively. These materials predominantly originated from Inner Mongolia and are the most suitable candidates as elite parental lines for dual-purpose breeding programs targeting simultaneous grain yield and forage biomass improvement.

Group II comprised 10 accessions (21.74%) distinguished by markedly shorter growth periods (76–90 days) and reduced plant height (105.74–139.32 cm), together with narrower stem diameters and compact panicle architecture. These early-maturing, semi-dwarf materials primarily originated from Xinjiang landraces and exhibited lower mean grain yields than Group I but demonstrated the most stable performance across years, attributed to their short-season phenology that avoids late-season heat and water stress. Their compact architecture confers excellent lodging resistance and mechanical harvestability, traits of direct practical value for modern proso millet production systems in northern Xinjiang.

Group III was the largest cluster, containing 26 accessions (56.52%) characterized by superior vegetative development, including the highest mean values for main stem height (150.88 cm), leaf length (25.10 cm), and leaf width (27.79 mm), as well as high total above-ground biomass. Despite modest grain yields, these materials produced the greatest vegetative biomass among the three groups, making them particularly suitable as foundation material for forage variety breeding and as sources of alleles governing vegetative vigor and stress tolerance. These accessions predominantly originated from Gansu, Ningxia, Shaanxi, Shanxi, and Hebei, reflecting the broad geographical provenance of the collection and the ecological diversity of their source environments. The clear separation of the three groups along both yield-component and phenological axes confirms that the germplasm collection encompasses complementary breeding pools that can be strategically combined, particularly Group I × Group II crosses to pyramid high yield with early maturity and lodging resistance.

### 3.8 Genotype × environment interaction analysis

Combined analysis of variance across the two growing seasons (2023 and 2024) revealed highly significant effects of genotype (G) for all twelve agronomic traits (*P < 0.001*), confirming substantial genetic variation in the germplasm collection (**Table 5**). The environmental main effect (year) was significant (*P < 0.05*) for leaf width, grain weight per panicle, thousand-grain weight, and yield, reflecting year-to-year climatic variation at the experimental site. Crucially, genotype × environment (G×E) interaction effects were non-significant for all traits, indicating that genotype rankings remained stable across years and that the expression of these agronomic traits was largely consistent under the irrigated conditions of northern Xinjiang.

**Table 5:** Analysis of Variance (ANOVA) for 12 quantitative traits of 46 proso millet germplasm accessions evaluated in 2023 and 2024..

Trait	Source of Variation	df	SS	MS	F	Sig.	H²
Growth Period (d)	Genotype (G)	45	17,765.739	394.794	1214.3	***	0.999
	Year (E)	1	0.000	0.000	0	ns	
	G×E	45	0.000	0.000	0	ns	
	Rep(E)	4	5.478	1.370	4.21	**	
	Error	180	58.522	0.325	—		
	Total	275	17,829.739		—		
No. of Productive Tillers	Genotype (G)	45	28.040	0.623	4.47	***	0.817
	Year (E)	1	0.000	0.000	0	ns	
	G×E	45	0.000	0.000	0	ns	
	Rep(E)	4	0.654	0.163	1.17	ns	
	Error	180	25.096	0.139	—		
	Total	275	53.790		—		
Main Stem Height (cm)	Genotype (G)	45	176,110.056	3,913.557	15095.61	***	0.941
	Year (E)	1	423.761	423.761	0.12	ns	
	G×E	45	11.666	0.259	0	ns	
	Rep(E)	4	14,434.587	3,608.647	14.65	***	
	Error	180	44,341.492	246.342	—		
	Total	275	235,321.562		—		
Main Panicle Length (cm)	Genotype (G)	45	6,838.225	151.961	12629.3	***	0.875
	Year (E)	1	16.952	16.952	0.06	ns	
	G×E	45	0.541	0.012	0	ns	
	Rep(E)	4	1,061.740	265.435	12.27	***	
	Error	180	3,893.134	21.629	—		
	Total	275	11,810.593		—		
No. of Main Stem Nodes	Genotype (G)	45	318.126	7.069	978.17	***	0.91
	Year (E)	1	16.800	16.800	2.59	ns	
	G×E	45	0.325	0.007	0.01	ns	
	Rep(E)	4	25.902	6.475	9.25	***	
	Error	180	126.033	0.700	—		
	Total	275	487.186		—		
Main Stem Diameter (mm)	Genotype (G)	45	326.277	7.251	517.01	***	0.91
	Year (E)	1	17.139	17.139	4.73	ns	
	G×E	45	0.631	0.014	0.02	ns	
	Rep(E)	4	14.501	3.625	5.05	***	
	Error	180	129.257	0.718	—		
	Total	275	487.805		—		
Leaf Length (cm)	Genotype (G)	45	3,217.881	71.508	6922.97	***	0.822
	Year (E)	1	16.337	16.337	0.06	ns	
	G×E	45	0.465	0.010	0	ns	
	Rep(E)	4	1,012.596	253.149	16.4	***	
	Error	180	2,778.512	15.436	—		
	Total	275	7,025.791		—		
Leaf Width (cm)	Genotype (G)	45	32.088	0.713	40.48	***	0.861
	Year (E)	1	13.346	13.346	39.86	**	
	G×E	45	0.793	0.018	0.16	ns	
	Rep(E)	4	1.339	0.335	2.99	*	
	Error	180	20.130	0.112	—		
	Total	275	67.696		—		
No. of Leaves	Genotype (G)	45	371.770	8.262	662.07	***	0.926
	Year (E)	1	17.840	17.840	7.52	ns	
	G×E	45	0.562	0.012	0.02	ns	
	Rep(E)	4	9.486	2.371	3.58	**	
	Error	180	119.398	0.663	—		
	Total	275	519.055		—		
Grain Weight per Panicle (g)	Genotype (G)	45	4,636.919	103.043	1615.36	***	0.996
	Year (E)	1	17.023	17.023	1813.35	***	
	G×E	45	2.871	0.064	0.15	ns	
	Rep(E)	4	0.038	0.009	0.02	ns	
	Error	180	74.153	0.412	—		
	Total	275	4,731.002		—		
1000-Grain Weight (g)	Genotype (G)	45	186.649	4.148	105.59	***	0.979
	Year (E)	1	16.360	16.360	681.13	***	
	G×E	45	1.768	0.039	0.45	ns	
	Rep(E)	4	0.096	0.024	0.28	ns	
	Error	180	15.571	0.087	—		
	Total	275	220.444		—		
Yield (kg·hm^-^²)	Genotype (G)	45	474,240.068	10,538.668	35758.28	***	0.998
	Year (E)	1	155.881	155.881	19.68	*	
	G×E	45	13.262	0.295	0.02	ns	
	Rep(E)	4	31.684	7.921	0.47	ns	
	Error	180	3,055.969	16.978	—		
	Total	275	477,496.864		—		

G, Genotype; E, Year (Environment); G×E, Genotype × Environment interaction; Rep (E), Replication nested within environment. σ²g, genotypic variance; σ²ge = G×E interaction variance; σ²e , residual (error) variance. H² = broad-sense heritability on a genotype-mean basis: H² = σ²g/(σ²g + σ²ge/e + σ²e/re), where e = 2 years, r = 3 replications. Significance codes: ***p <no><</no> 0.001; **p <no><</no> 0.01; *p <no><</no> 0.05; ns = not significant (*p > 0.05*). F-value for Genotype (G) is tested against G×E mean square; F for Year (E) is tested against Rep (E) mean square.

Broad-sense heritability estimates were very high for most traits. Grain weight per panicle exhibited the highest heritability (H² = 0.996), followed by grain yield (H² = 0.998) and thousand-grain weight (H² = 0.979), indicating that these traits are predominantly under genetic control and are highly reliable targets for phenotypic selection. High heritability was also estimated for growth period (H² = 0.999), main stem height (H² = 0.941), main panicle length (H² = 0.875) and main stem diameter (H² = 0.910) (**Supplementary Table 2**). These heritability estimates support the use of mean performance across environments as a reliable selection criterion for yield-related traits in northern Xinjiang conditions. However, it is important to note that the trial was conducted under irrigated conditions (four uniform irrigations during the growth cycle); therefore, these heritability estimates may not be directly applicable to dryland or rainfed production environments where water stress could amplify G×E interaction effects and reduce the proportion of variance attributable to genotype.

Inter-annual comparison revealed that 2024 was characterized by higher average temperatures and lower precipitation during the grain-filling period compared to 2023, resulting in a mean yield reduction of approximately 6.8% in 2024 relative to 2023. Despite this environmental variation, Group I accessions maintained superior yield performance across both seasons, confirming their stability and suitability as elite parental materials. Several accessions from Group II exhibited notably improved relative performance in 2024, suggesting greater adaptation to warmer, drier growing conditions consistent with their short-season, drought-tolerant phenotype.

### 3.9 Regression analysis of yield-contributing traits

Stepwise multiple regression analysis identified the key agronomic traits contributing to grain yield variation. Grain weight per panicle (GWP) entered the model first as the single most significant predictor (R² = 0.880, *P < 0.001*), followed by main panicle length (MPL), which significantly improved model fit (R² = 0.910, *P < 0.001*). No additional variables met the entry criterion (*P ≤ 0.05*), indicating that GWP and MPL together account for the majority of yield variation in this germplasm. The final regression equation was:


Y = –2148.3 + 98.6 × GWP + 52.4 × MPL

where Y is grain yield (kg·ha^-^¹), GWP is grain weight per panicle (g), and MPL is main panicle length (cm). The model demonstrated high predictive accuracy (adjusted R² = 0.906; standard error of estimate = 187.3 kg·ha^-^¹), indicating that approximately 91% of grain yield variation among accessions can be reliably predicted from these two panicle-related traits alone.

Standardized regression coefficients confirmed that GWP (β = 0.621) exerted a substantially greater direct effect on yield than MPL (β = 0.298), consistent with the higher bivariate correlation between Y and GWP and confirming grain weight per panicle as the dominant yield determinant. The practical implication of this model is that breeders can apply indirect selection for yield through direct measurement of GWP and MPL-traits that are easier to measure early in the season and with higher precision than plot-based yield harvests.

## 4 Discussion

### 4.1 Genetic diversity patterns

The 46 proso millet accessions evaluated in this study exhibited substantial phenotypic diversity across both qualitative and quantitative traits, demonstrating the breadth of variation available within Chinese proso millet germplasm for breeding applications. The higher Shannon diversity indices for grain color (H′ = 2.064) and panicle branch length (H′ = 2.045) relative to panicle type (H′ = 1.383; **Figure 2**) suggest that these traits have experienced less directional selection pressure and retain broader allelic variation, consistent with previous reports on proso millet germplasm diversity in China (Sharma and Niranjan, 2018; Zhang et al., 2023). The predominance of yellow grain color (86.96%; **Figure 3**) aligns with distribution patterns documented in northern Chinese germplasm collections (Nandini et al., 2026), reflecting both strong consumer market preferences and the ecological adaptation of cultivated types to regional agricultural systems. Similarly, the high frequency of lateral panicle types (71.74%) and long panicle branches (63.04%; **Figure 3**) is consistent with the morphological adaptation of high-latitude proso millet germplasm (Vetriventhan et al., 2019), where maximizing panicle surface area supports efficient grain filling under the relatively short growing seasons of northern China.

Among quantitative traits, leaf width exhibited the highest coefficient of variation (CV = 24.14%; **Table 2**), substantially exceeding all other traits including yield-related characters. This high variability likely reflects the sensitivity of leaf width to both genotypic differences and microenvironmental variation during vegetative growth, and suggests that leaf width may be a particularly responsive indicator of genotype × environment interactions in this germplasm. Grain yield and grain weight per panicle also showed considerable variation (CV = 15.00% and 14.61%, respectively), corroborating findings from He et al. (2015) and Lv et al. (2025) and confirming that meaningful yield improvement through phenotypic selection remains feasible within this collection. In contrast to the CV pattern, leaf length demonstrated the highest Shannon diversity index of all quantitative traits (H′ = 2.155; **Table 2**), suggesting that while the absolute range of leaf length variation may be moderate, accessions are broadly distributed across phenotypic classes, a pattern indicative of diverse genetic backgrounds rather than clustering around a single adaptive optimum. The relatively stable growth period across accessions (CV = 8.23%) reflects the phenological adaptation of these cultivated varieties to the specific photoperiod and thermal conditions of their respective regions of origin, consistent with observations in other temperate cereal crops (Ma et al., 2020). Taken together, these diversity patterns indicate that the evaluated accessions capture a broad range of phenotypic variation suitable for identifying superior genotypes for targeted breeding programs.

### 4.2 Trait correlations and yield formation

The strong positive correlations between grain yield and both grain weight per panicle (r = 0.901, *P < 0.01*) and main panicle length (r = 0.863, *P < 0.01*) (**Figure 5**), confirm the ‘large panicle, high yield’ strategy as the dominant yield-formation pathway in this germplasm, and demonstrate that panicle-based traits are the most actionable selection criteria for yield improvement under northern Xinjiang conditions. This finding aligns with Lv et al. (2025), who identified panicle length as a key trait with the highest loading in the first principal component in a phenotypic diversity study of proso millet germplasm from Xinjiang and neighboring regions, and is consistent with yield component analyses reported for other small-grain cereals (Du et al., 2020; Zhang et al., 2025; Zhou et al., 2025), where panicle architecture is the primary determinant of sink capacity. These relationships are further supported by the stepwise regression model, which explained 91% of yield variation using only two panicle traits (GWP and MPL), providing a highly efficient and practically deployable indirect selection strategy for breeding programs (Wei et al., 2024). The strong positive correlation between thousand-grain weight and grain yield (r = 0.847, *P < 0.01*) (**Figure 5**) further supports a dual strategy of simultaneously improving panicle size and individual grain filling to maximize yield gains. Main stem height exhibited strong positive correlations with main stem node number and main stem diameter (r ≥ 0.884, *P < 0.01*) but showed a weak positive correlation with productive tillers (r = 0.195, not significant) (**Figure 5**), reflecting a plant architecture that prioritizes main stem growth without a pronounced trade-off with tillering-an adaptive strategy for concentrating photoassimilates in the main panicle under semi-arid, high-irradiance conditions where source-limited grain filling is a primary constraint. This pattern aligns with reports documenting resource allocation strategies in drought-stressed cereals (Stevens et al., 2021).

### 4.3 Genotype × environment interactions and heritability

The non-significant genotype × environment (G×E) interaction effects detected for all agronomic traits (**Table 5**) indicate that genotype rankings remained stable across the two growing seasons (2023 and 2024), highlighting the consistency of phenotypic expression under the irrigated conditions of northern Xinjiang. This stability is particularly valuable for breeding programs, as it suggests that selection decisions based on single-year trials can be reliably applied across environments, provided irrigation remains consistent.

The very high broad-sense heritability estimates for yield-related traits, grain weight per panicle (H² = 0.996), grain yield (H² = 0.998) and thousand-grain weight (H² = 0.979), indicate that these traits are predominantly under genetic control and are highly reliable targets for phenotypic selection. High heritability was also observed for growth period (H² = 0.999), main stem height (H² = 0.941), main panicle length (H² = 0.875), and main stem diameter (H² = 0.910). These values substantially exceed the moderate heritability estimates commonly reported in rainfed cereal trials (Savin et al., 2022), reflecting the favorable irrigated conditions that minimized environmental noise and maximized genetic expression. The consistent superiority of Group I accessions across environments further validates their potential as stable, high-yielding parental materials for northern Xinjiang breeding programs. It should be noted, however, that all trials were conducted under irrigated conditions with four standardized irrigation events; thus, the reported heritability values reflect the genetic contribution to phenotypic variation in the absence of water deficit. Genotype rankings and heritability estimates may differ under dryland or rainfed conditions where G×E interactions driven by water availability could be substantially larger. Future multi-location, multi-environment trials including dryland settings are recommended to validate the stability of these findings for broader application.

### 4.4 Germplasm structure and breeding implications

The cluster analysis (**Figure 6**) revealed three phenotypically and geographically distinct groups that reflect the ecological differentiation of Chinese proso millet germplasm resources across diverse agro-climatic zones, consistent with the geographic structure of genetic diversity documented by Wang et al. (2021) and Lv et al. (2025).

Group I materials originating predominantly from Inner Mongolia demonstrated superior yield components grain yield, grain weight per panicle, and thousand-grain weight all significantly exceeding population means representing the highest-priority candidates for use as elite parental lines in yield-focused breeding programs.

Group II materials from Xinjiang, characterized by shorter growth periods (76–90 days) and semi-dwarf stature (105.74–139.32 cm), are ideally suited for early-maturing cultivar development adapted to high-latitude or double-cropping production systems (Mude et al., 2020). Their compact, mechanically harvestable plant architecture and stable cross-year performance make them particularly valuable for developing commercial varieties compatible with large-scale mechanized production in Xinjiang.

Group III materials, originating from diverse southern and central northern provinces (Gansu, Ningxia, Shaanxi, Shanxi, and Hebei) and characterized by vigorous vegetative development, offer valuable germplasm resources for forage variety breeding, biomass-oriented programs, and as a source of alleles governing stress-adaptive vegetative traits.

Strategic crossing between groups, particularly Group I × Group II combinations to pyramid high yield with early maturity and lodging resistance, and Group I × Group III combinations to introduce biomass-associated traits into high-yield backgrounds represents the most promising near-term avenue for variety development in northern Xinjiang. To further validate and accelerate these breeding strategies, future research should incorporate molecular marker characterization, including SSR and SNP-based genomic tools, to complement phenotypic evaluation, identify QTL associated with key yield traits, facilitate marker-assisted selection, and broaden the genetic base through targeted exotic germplasm introduction (Tyagi et al., 2024, 2025; Lv et al., 2025; Wang et al., 2017; Yang et al., 2025; Yin et al., 2024). Multi-environment trials under dryland and irrigated conditions across additional locations in northern Xinjiang are also strongly recommended to assess genotype stability and refine selection criteria for broader regional deployment.

## 5 Conclusion

This study systematically evaluated 46 proso millet accessions sourced from seven provinces across two growing seasons (2023–2024) in northern Xinjiang, revealing substantial and actionable genetic diversity at both qualitative and quantitative trait levels. Among qualitative traits, grain color (H′ = 2.064) and panicle branch length (H′ = 2.045) exhibited the highest genetic diversity, while panicle type showed the lowest (H′ = 1.383). Among quantitative traits, grain yield (CV = 15.00%) and grain weight per panicle (CV = 14.61%, H′ = 2.122) showed the greatest variation, confirming substantial potential for yield improvement through targeted phenotypic selection. The diversity indices for these complex traits should be interpreted in the context of the non-linear nature of the Shannon–Weaver index and the geographic structure of the collection, wherein approximately 60% of accessions originate from three provinces (Inner Mongolia, Gansu, and Xinjiang).

Combined analysis of variance confirmed highly significant genotypic effects for all agronomic traits, while genotype × environment interaction effects were non-significant for all traits, indicating stable genotype rankings across the two growing seasons under irrigated conditions. Very high broad-sense heritability estimates were observed for yield-related traits grain weight per panicle (H² = 0.996), grain yield (H² = 0.998) and thousand-grain weight (H² = 0.979), demonstrating that these traits are predominantly under genetic control and are highly reliable targets for phenotypic selection under northern Xinjiang conditions. It should be noted, however, that these estimates reflect performance under irrigated management and may not fully extend to dryland or rainfed environments.

Stepwise regression analysis established a highly predictive yield model (R² = 0.91) using grain weight per panicle (β = 0.621) and main panicle length (β = 0.298) as the sole predictors, providing a practical, early-season indirect selection criterion that reduces reliance on laborious plot-level yield harvests.

Cluster analysis identified three functionally distinct germplasm groups: Group I (21.74%; predominantly from Inner Mongolia) with superior yield performance (mean 5,112.90 kg·ha^-^¹), suitable as elite parental lines for dual-purpose breeding; Group II (21.74%; predominantly Xinjiang landraces) with early maturity (76–90 days), compact architecture (105.74–139.32 cm) and stable cross-environment performance, ideal for developing short-season cultivars adapted to mechanized harvesting; and Group III (56.52%; predominantly from Gansu, Ningxia, Shaanxi, Shanxi, and Hebei) with superior vegetative biomass, valuable for forage breeding and as a repository of stress-adaptive alleles. Strategic crossing among groups, particularly Group I × Group II-offers the most promising near-term route to combining high yield potential with early maturity and lodging resistance. These findings provide an empirical foundation and prioritized germplasm resources for evidence-based parent selection and targeted breeding program design in support of sustainable and productive proso millet cultivation in northern Xinjiang.

## Data Availability

The original contributions presented in the study are included in the article/**Supplementary Material**. Further inquiries can be directed to the corresponding authors.
